# The Role of the Thalamus in Nociception: Important but Forgotten

**DOI:** 10.3390/brainsci14080741

**Published:** 2024-07-25

**Authors:** Giovane Galdino, Flavio Protasio Veras, Tayllon dos Anjos-Garcia

**Affiliations:** Multicenter Postgraduate Program in Physiological Sciences, Department of Physiological Sciences, Institute of Biomedical Sciences, Federal University of Alfenas, Alfenas 37133-840, MG, Brazil

**Keywords:** pain, thalamus, glial cells

## Abstract

Pain is a complex response to noxious stimuli. Upon detection of the nociceptive stimulus by first-order neurons or nociceptors, an action potential ascends to the spinal dorsal horn, a crucial site for synapsing with second-order neurons. These second-order neurons carry the nociceptive stimulus to supraspinal regions, notably the thalamus. Although extensive research has focused on spinal-level nociceptive mechanisms (e.g., neurotransmitters, receptors, and glial cells), the thalamus is still poorly elucidated. The role of the thalamus in relaying sensory and motor responses to the cortex is well known. However, a comprehensive understanding of the mechanisms in the synapse between the second-order and third-order neurons that transmit this impulse to the somatosensory cortex, where the response is processed and interpreted as pain, is still lacking. Thus, this review investigated the thalamus’s role in transmitting nociceptive impulses. Current evidence indicates the involvement of the neurotransmitters glutamate and serotonin, along with NMDA, P2X4, TLR4, FGR, and NLRP3 receptors, as well as signaling pathways including ERK, P38, NF-κB, cytokines, and glial cells at nociceptive synapses within the thalamus.

## 1. Introduction

The function of pain in the human body is essential. Its role is to alert the occurrence of a potentially harmful stimulus or an actual injury, as well as to promote appropriate behavioral responses to avoid such harmful stimuli [1]. The International Association for the Study of Pain (IASP) describes pain as an unpleasant sensory and emotional experience associated with or similar to that resulting from potential or actual damage [2]. However, chronic pain can lose its protective character due to changes in the nociceptive pathway, significantly affecting the individual’s quality of life [3].

The pain can be acute, which is characterized by sudden and short-term discomfort, disappearing when the injury and its cause are resolved. However, the pain becomes chronic if it persists for more than 3 months with or without the triggering stimulus. Chronic pain is characterized by complex peripheral and central processes that contribute to its maintenance, among which neuroimmune interactions stand out. Among the types of chronic pain, those caused by neuropathy and osteoarthritis affect a significant proportion of the world’s population [2,3,4].

Particularly, this review focuses on neuropathic pain (NP), which is among the most difficult to control. Most available treatments for this type of pain have moderate efficacy, in addition to presenting side effects that limit their use, significantly reducing the quality of life of individuals suffering from this condition [4]. It is estimated that NP affects between 3% and 17% of the world’s population [5], mainly originating from physiological conditions and states such as metabolic disorders (e.g., peripheral diabetic neuropathy), neuropathies associated with viral infections (e.g., post-herpetic neuralgia, HIV, and leprosy), autoimmune disorders affecting the central nervous system (e.g., multiple sclerosis and Guillain–Barré syndrome), chemotherapy-induced peripheral neuropathies, traumatic damage to the nervous system (e.g., trauma in peripheral nerves, spinal cord injury, and amputation), inflammatory disorders, hereditary neuropathies, and channelopathies [4].

In addition to being resistant to different treatment modalities, diagnosing neuropathic pain is not a simple task. Accordingly, the diagnosis includes a rigorous anamnesis and adequate physical examination. Furthermore, quantitative sensory tests can be used, which are psychophysical techniques that measure the perception of controlled cutaneous stimuli of ascending and descending magnitude in patients with suspected neuropathic pain. Neurophysiological techniques that include nerve conduction studies, measurement of somatosensory evoked potentials, trigeminal reflexes, laser-evoked potentials, contact-heat evoked potentials, and microneurography are also relevant options for diagnosing pain. Finally, immunostaining in skin biopsies allows for determining intraepidermal nerve fiber density, while magnetic resonance imaging can be used to assess the anatomical integrity of brain regions of nociceptive processing during neuropathic pain [6,7]. Thus, these methods may be useful in the early detection of neuropathic pain.

The number of studies aiming to elucidate the mechanisms involved in NP has increased in recent years; these are mainly focused on the discovery of target molecules and structures involved in its nociceptive pathway with potential relevance for the development of new treatments for its control. Thus, this review focused on the role of glial cells and neuroimmune interactions within the thalamic nuclei in the genesis of pain, with emphasis on the cellular and molecular mechanisms involved in this process.

## 2. Pain Transmission Pathway

Before understanding pain transmission, it is important to know that pain is classified into three main types—nociceptive, neuropathic, and neoplastic: (I) nociceptive pain caused by real or threatened tissue damage, (II) neuropathic pain caused by an injury or disease of the somatosensorial system, and (III) neoplastic pain that arises from altered nociception in the absence of tissue or nervous system damage [8]. Physiologically, pain originates when a nociceptive stimulus occurs, activating specific receptors present in different areas of our body, such as the skin, nerves, mucous membranes, muscles, joints, and viscera [2]. These stimuli are detected by the free nerve endings of first-order afferent neurons, also known as nociceptors.

Nociceptors will conduct the nociceptive impulse to the superficial layers of the spinal dorsal horn through Aδ, Aβ, and C fibers [4]. Aδ fibers are medium-sized, thinly myelinated, and have a conduction velocity of the nociceptive impulse between 20 and 30 m/s [9]. On the other hand, C fibers have a small diameter and are unmyelinated, resulting in a slow impulse velocity (0.5 to 2.0 m/s) [9]. Aδ fibers respond to mechanical and thermal stimuli, while C fibers respond to mechanical, thermal, and chemical stimuli, being termed polymodal [9].

Once mechanical, thermal, or chemical stimuli are detected by nociceptors, the impulse is then conducted by the primary afferent fibers and enters the spinal dorsal horn, where they will make a first synapse with second-order neurons in laminae I and II of the ipsilateral spinal dorsal horn for sensations arising on the body surface or the spinal trigeminal nucleus for sensations on the head [10]. The second-order neurons are responsible for conducting the impulse mainly to the thalamus but also to the reticular formation and mesencephalon. Classical neurotransmitters such as glutamate and neuromodulators such as substance P and calcitonin gene-related peptide (CGRP) are involved in this process, which is released by the first-order neuron [11]. After synapsing at the spinal dorsal horn, the second-order neurons decussate in the spinal cord and ascend in the anterolateral funiculus, leading impulses through the tracts of the anterolateral system (e.g., spinothalamic, spinoreticular, and spinomesencephalic tracts) and reaching primarily the brainstem and thalamus [11]. The axons of the spinothalamic tract for sensation arising on the body ascend in the ventral (anterior) funiculus and end in the ventral posterolateral nucleus (VPL) of the thalamic ventrobasal complex. On the other hand, the first-order neurons for sensation on the head enter the brainstem through the trigeminal nerve, and the axons descend via the spinal trigeminal tract to the spinal trigeminal nucleus, which makes a first synapse with second-order neurons that ascend (a trigeminothalamic tract) collaterally and/or contralaterally to the ventral posteromedial nucleus (VPM) of the thalamic ventrobasal complex. In both cases, these components are related to the discriminative and sensory aspects of pain [12,13].

Meanwhile, the axon bundle of the spinoreticular tract ascends contralaterally and terminates in nuclei of the brainstem reticular formation, where they synapse with a third neuron that reaches the intralaminar nuclei of the thalamus, and then another neuron achieves the various anatomical regions of the cerebral cortex involved in the affective–cognitive component of pain. Similarly, the spinomesencephalic tract reaches mesencephalic nuclei, such as periaqueductal gray matter, which are important nuclei involved in endogenous pain control as well as motivational aspects of pain [14,15].

**Figure 1:**
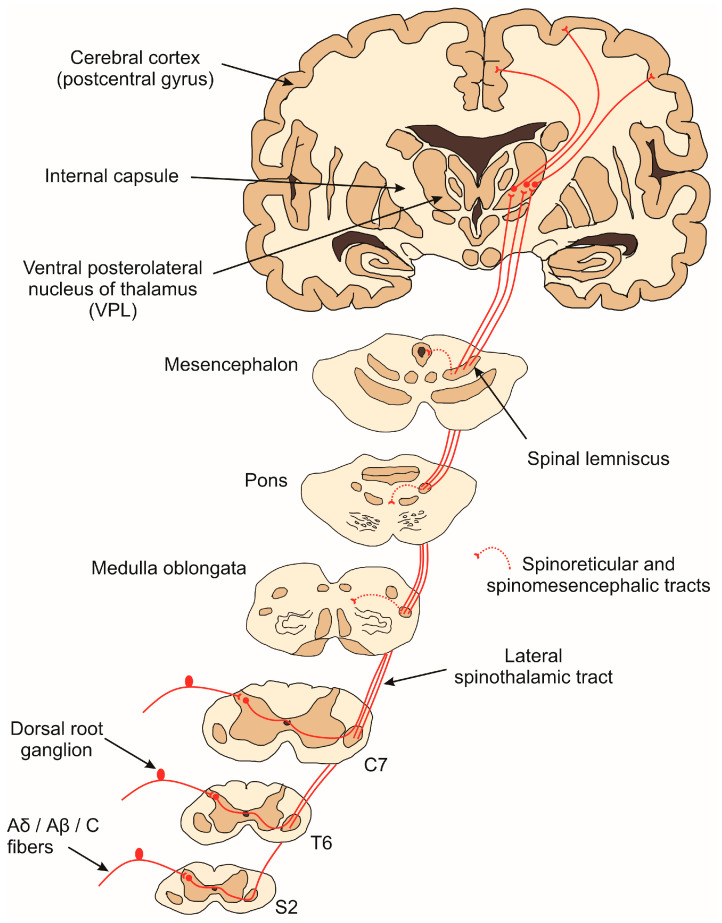
Pain pathway. Pain, temperature, and pressure sensations below the head are transmitted to the primary somatosensory cortex (postcentral gyrus) by the anterolateral system (spinothalamic and spinoreticular tracts).

In the thalamus, the third-order neurons then conduct the nociceptive impulse to the ipsilateral postcentral gyrus (primary somatosensory cortex) through the internal capsule, where it will be processed and interpreted as pain [16]. A schematic representation of the pain pathway can be visualized in Figure 1. Although the thalamus is a region of remarkable functional relevance, acting as a relay station for the main sensory systems, the mechanisms involved in this process have been poorly elucidated compared to other regions, such as the spinal dorsal horn.

## 3. The Thalamus and Nociception

The thalamus is an ovoid-shaped structure located in the diencephalon between the cerebral cortex and the midbrain, primarily composed of gray matter (neuronal nuclei) in the brain [17]. It consists of two neuronal masses located in the central region of each cerebral hemisphere, connected by an intermediate structure called an interthalamic adhesion [17]. The thalamus plays a crucial role in physiology, serving as a relay station for major sensory systems (except the olfactory pathway) [18]. Additionally, the thalamus is implicated in motor control (extrapyramidal), emotional behavior, and the level of cortical activation [18]. Thus, thalamic activities are closely related to the cerebral cortex.

Anatomically, each thalamus is primarily composed of gray matter, divided by a Y-shaped white matter structure called the internal medullary lamina. The thalamus’s functions are largely determined by its nuclei, which are divided into three main groups by the internal medullary lamina: anterior, lateral, and medial groups [19]. The lateral group is further subdivided into posterior, dorsal, and ventral subgroups. In addition to these three main groups, the reticular nucleus of the thalamus and the medial group stand out [19]. Together, these groups form the sixteen nuclei found in the thalamus, with the majority located in its lateral part.

**Figure 2:**
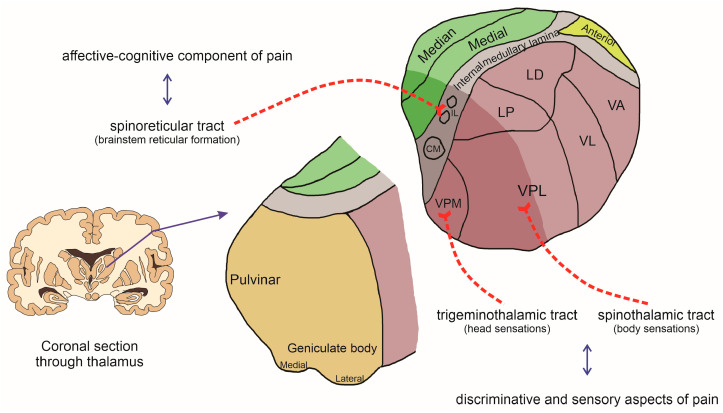
Schematic section through thalamus with a focus on the ascending pathways ultimately conveyed in thalamic nuclei involved in the affective–cognitive, discriminative, and sensory aspects of pain. Thalamic nuclei: IL (intralaminar nuclei), CM (centromedian), LP (lateral posterior), LD (lateral dorsal), VA (ventral anterior), VL (ventral lateral), VPL (ventral posterolateral), and VPM (vental posteromedial).

As described previously, the thalamic ventrobasal complex (VB), composed of the VPL and VPM nuclei (Figure 2), receives spinothalamic projections from the spinal dorsal horn and is involved in the discriminative and sensory aspects of pain processing [12,13]. The VPM nucleus is involved in somatosensation of the head area, receiving information from the trigeminal nerve complex (nuclei of the V cranial nerve) [12,13]. Conversely, the VPL nucleus is involved in somatosensation of the body (trunk and extremities). It receives information from the medial lemniscus pathway (signaling light touch and proprioception) and the spinothalamic tract (signaling temperature and painful stimuli), projecting to the primary somatosensory cortex [12,13].

In the mid-1980s, studies conducted by Guilbaud et al. highlighted the thalamus’s contribution to hyperalgesic responses (increased painful response to noxious stimuli) associated with peripheral lesions [20,21,22,23]. These studies demonstrated that following hind paw inflammation or sciatic nerve injury in rats, ventrobasal thalamic neurons exhibited reduced thresholds and enhanced peripheral-evoked responses. However, the authors did not specify which VPM or VPL subnuclei participated in this response. Before these studies, another study had shown that spinal cord injury in the dorsal and ventral horns resulted in extensive thalamic atrophy, with the VPM nucleus being particularly affected [24]. Thus, these findings indicated that the thalamus, particularly the VPM and VPL nuclei, is involved in the relay of nociceptive impulses, originating mainly from the dorsal horn of the spinal cord to the somatosensory cortex. However, the cellular and molecular mechanisms involved in thalamic pain physiology are not well elucidated.

Voltage-gated sodium channels (Nav) are well-known in the nociceptive process for their ability to generate and conduct action potentials along the axonal membrane of first-, second-, and third-order neurons [25]. In this context, Na_v_ involvement was one of the first mechanisms investigated in the increased nociceptive impulse in the thalamus, particularly Na_v_1.3 in different experimental pain models. Initial studies showed Na_v_1.3 expression upregulation in VPL and VPM nuclei 4 weeks after spinal cord injury, which was associated with increased spontaneous neuronal discharge in these regions, as well as allodynia and hyperalgesia [26,27]. Another study found that 10 days after neuropathic pain induced by a sciatic nerve constriction model, there was an increase in Na_v_ mRNA expression in neurons of the contralateral VPL nucleus contralateral to the constricted limb [28].

Studies have also identified the involvement of N-methyl-D-aspartate (NMDA) receptors, an ionotropic glutamate-activated receptor, in somatosensory and nociceptive transmission in the thalamus [29,30]. A previous study with in vivo electrophysiological recordings found increased synaptic density in sensory neurons of the ventrobasal complex of the thalamus in rats after vibrissa stimulation with an air jet or glutamatergic agonist administration. This effect was reversed by CPP, a selective NMDA receptor antagonist [29]. Subsequently, Dougherty et al. [30] showed increased neuronal activity in the VPL nucleus after glutamatergic agonist administration at this site or nociceptive stimuli applied to the skin. Furthermore, this effect was reversed by AP7 pretreatment, a specific NMDA receptor antagonist [30]. NMDA receptors in the thalamus are also involved with nociception induced in a rat paw inflammation model. This was verified by intra-VPL nucleus injection of the NMDA receptor antagonist D,2-amino-5-phosphonovaleric acid (D-APV), which reversed the acute thermal and mechanical hyperalgesia induced by hindlimb intraplantar carrageenan injection. In addition, pretreatment with the NR1 subunit of the NMDA receptor antisense oligodeoxynucleotides prevented hyperalgesia and decreased the number of thalamic NMDA receptors [31].

In addition to inflammation-induced nociception, nociceptive neuron activity in the VPL nucleus of the thalamus was increased during peripheral neuropathy induced by chronic constriction injury of the sciatic nerve, and pretreatment with the NMDA receptor antagonist MK801 suppressed noxious stimulus-evoked activity in VPL neurons [32].

Supporting previous findings, microdialysis experiments found increased glutamate levels in the VPL nucleus following carrageenan-induced paw inflammation in rats [33]. Glutamate is the most abundant amino acid in the central nervous system (CNS), and these studies have demonstrated its importance in neuronal excitability in the VPL nucleus of the thalamus via NMDA receptors during nociception transmission.

The metabotropic glutamate receptor type 1 (mGlu1) is also involved in this process. Studies conducted by Salt et al. [34,35] demonstrated that (*S*)-3,5-dihydroxy-phenylalanine administration, a mGlu1 receptor agonist in the ventrobasal complex of the thalamus, increased neuron excitability in this region in response to a thermal or electronically gated air jet nociceptive stimulus applied to the contralateral paw or vibrissae in rats, respectively. This effect was potentiated by the mGlu1 receptor-positive allosteric modulator Ro67-4853 and reversed by LY367385 pretreatment, an antagonist of these receptors [34,35].

Evidence also demonstrates that the serotonergic system plays an important role in regulating chronic pain at the brain level [36]. An interesting study found a significant reduction in serotonin (5-HT) levels in the ventrobasal complex of the thalamus in rats subjected to neuropathic pain induced by sciatic nerve constriction [37]. In addition, 5-HT levels in dialysates from the contralateral ventrobasal thalamus were significantly lower in rats with neuropathy compared to sham animals. The authors suggested that the reduction in this neuromodulator during neuropathy might be related to a decrease in its action on the spinal cord neuronal projections entering the thalamus. It has also been demonstrated that 5-HT receptors participate in nociception transmission in the thalamus. Studies conducted by Xiao et al. [38,39] found that radiant-heat-evoked tail flick was reversed by 5-HT microinjection into the thalamic ventrobasal complex. Furthermore, this effect was prevented by 5-HT_1A_ and 5-HT_2_ receptor antagonist administration but not by 5-HT_3_ receptor antagonists. 

In addition to modulating the nociceptive impulse in the thalamus, 5-HT via thalamic projections also modulates the nociceptive synapse in the spinal cord. A previous study found that electrical stimulation of the thalamic ventrobasal complex reduced C-fiber activity in spinal dorsal horn neurons evoked by noxious cutaneous electrical stimulation in rats [40]. Like 5-HT, opioid receptors can modulate nociceptive impulses in the thalamus. A study found that intravenous naloxone administration, a non-selective opioid receptor antagonist, reduced evoked stimuli in VPM nucleus neurons during electrical stimulation of the sural nerve in rats [41].

Although the studies presented previously have demonstrated some mechanisms involved in the transmission of nociceptive impulses at the thalamus level, recent evidence suggests that this process may be more complex and involves neuroimmune interactions similar to that found at the spinal level [42,43,44]. Depending on the stimulus and type of pain, this neuroimmune interaction may be involved in pain chronification, changing the response from physiological to pathological. This process may be involved in central pain sensitization.

## 4. Chronic Pain and Central Pain Sensitization: Likely Involvement of the Thalamus

Chronic pain can affect up to 20% of the global population and is defined by the International Association for the Study of Pain (IASP) as persistent or recurrent pain lasting more than 3 months [2,3,4]. It can be disabling, leading to psychosomatic problems, occupational withdrawal, and a significant reduction in quality of life [3,4,5] Although some mechanisms involved in chronic pain have been described, the treatment may still be refractory in many cases, emphasizing the importance of further studies.

Unlike peripheral sensitization, central sensitization results from changes in the properties of CNS neurons. These neuronal changes, associated with glial cell activation, alter CNS responses to sensory stimuli rather than simply reflecting the presence of peripheral noxious stimuli. As a result, there is an increased response to noxious or innocuous painful stimuli, even after the initial cause has disappeared or when no peripheral pathology is present [44,45,46,47].

An additional and important phenomenon that supports the pain-protective function is the sensitization of the nociceptive system, which occurs after repeated or particularly intense noxious stimuli, which may result in pain chronification [42,44,45,47]. In this process, the threshold for activation decreases, and responses to subsequent inputs are amplified. In the absence of ongoing tissue damage, this state of increased sensitivity returns over time to the normal baseline, where high-intensity stimuli are again required to initiate pain. This is a long-lasting but not permanent phenomenon [16]. However, pain is no longer protective in some clinical situations, emerging spontaneously or being triggered by normally innocuous stimuli (allodynia) or exaggerated in response to noxious stimuli (hyperalgesia). This kind of pain can be long-lasting, as in the case of chronic pain (lasting for months), and extends beyond the injury site (secondary hyperalgesia) [43]. These changes characterize central pain sensitization, defined as an amplification of neuronal signaling within the central nervous system (CNS) that induces pain hypersensitivity. In other words, an increased response of nociceptive afferent neurons in the CNS compared to their normal or subliminal input [42,44].

In the last decade, studies have demonstrated the importance of the neuroimmune response in the development and maintenance of central sensitization and, consequently, chronic pain [45,47,48]. When central sensitization was first described in the early 1980s, it was believed to be restricted to neurons. However, strong evidence has shown the involvement of glial cells in this process [45,47,48].

## 5. Mechanisms Involved in Central Pain Sensitization via Glial Cells

Glial cells constitute about 70% of the CNS cells, outnumbering neurons. In addition, previous studies using specific microglia and astrocyte markers demonstrated that these cells are widely distributed in the thalamus [49]. Although each different type of glial cell plays its role according to its characteristics, they were historically considered support cells responsible only for filling spaces in the CNS. The term “glia” is derived from the Greek, meaning “glue”, suggesting that these cells were seen merely as a structure to support neurons [50]. However, it is now known that glial cells not only provide support and maintain neuronal function but can also interact with neurons to modulate their excitability [51].

Glial cells in the CNS include astrocytes, microglia, and oligodendrocytes. Astrocytes are the most predominant glial cells in the CNS, playing metabolic, structural, homeostatic, and neuroprotective functions, such as controlling neurotransmitter levels, stabilizing and regulating the blood–brain barrier, and promoting synapse formation [51,52]. Microglia represent the immune-competent cells of the CNS, exhibiting phagocytic capabilities and “patrolling” the neuronal environment against potential threats [51]. Oligodendrocytes are responsible for myelinating the axons of CNS neurons, a crucial function that provides these axons with saltatory conduction, favoring the speed of action potential transmission. Despite their role in maintaining a healthy environment in the CNS under normal conditions, recent evidence has highlighted the role of glial cells in modulating the nociceptive pathway [53].

In addition to expressing receptors for the major excitatory neurotransmitters of the nociceptive pathway [54], such as the “classics” mentioned earlier, glial cells also express receptors for other signaling molecules released by peripheral nociceptors. For example, the neuromodulator calcitonin gene-related peptide released by nociceptors in the spinal dorsal horn has been shown to cause microgliosis (microglial cell proliferation and hypertrophy), a process associated with mechanical allodynia [55]. Another example involves nociceptors expressing chemokines, such as fractalkine (CX3CL1), which can lead to microglial activation via the activation of its receptor CX3CR1, resulting in intracellular phosphorylation of p38 mitogen-activated protein kinase (MAPK) during neuropathic pain [56]. Thus, molecules released by peripheral neurons can activate glial cells. The chemokine CCL2 is also implicated in astroglial–neuronal signaling following nerve injury in mice [57,58]. This process in microglia is quite contradictory [59], although a previous study demonstrated *Ccl2* gene expression in intervertebral disc samples from patients diagnosed with chronic spinal compression and demonstrated that CCL2 depletion in microglia mitigated chronic pain severity in mice [60]. However, additional evidence is still needed to demonstrate CCL2 involvement in neuron–microglia crosstalk in central nociception.

Specifically, CCL2 rapidly induces central sensitization by increasing the activity of NMDA glutamate receptors in second-order neurons of the spinal dorsal horn [61]. In addition to chemokines, purinergic receptors are involved in glial activation. Studies have found that adenosine type 2A (A_2A_) and ionotropic purinergic type 2X4 (P2X4) and type 2X7 (P2X7) receptors can activate microglia and astrocytes, contributing to central pain sensitization [62].

Glial cells also possess pattern recognition receptors (PRRs), such as toll-like receptors (TLRs), which detect pathogen-associated molecular patterns (PAMPs) or damage-associated molecular patterns (DAMPs). It has been demonstrated that toll-like receptor 4 (TLR4) is expressed in microglia and plays a significant role in painful hypersensitivity in animal models [63]. In neuropathic pain, the detection of these patterns by TLRs activates intracellular cascades that lead to the activation of genes responsible for the synthesis and release of pro-inflammatory mediators. Among the intracellular signaling pathways involved in this process, the mitogen-activated protein kinase (MAPK) pathway has received attention [46]. The stimulation of this pathway often results in the activation of nuclear transcription factors such as NF-κB, which loses its inhibitory subunit, translocates to the nucleus, and activates the gene machinery related to the production of pro-inflammatory mediators, such as IL-1β, IL-6, and TNF-α [58]. Finally, pro-inflammatory cytokines released by glial cells can activate quiescent microglia and astrocytes in a paracrine manner, creating a positive feedback loop and recruiting more glial cells [58]. Moreover, there is crosstalk between microglia and astrocytes, in which one cell type can activate the other, enhancing the release of these mediators [64].

To amplify neuronal excitability, cytokines also activate second-order neurons. Studies have shown that once activated, cytokine receptors activate intracellular second messengers such as ERK kinase, increasing the conductivity of certain receptors for excitatory neurotransmitters, such as glutamatergic AMPA and NMDA receptors [65]. Thus, glial cells can influence the excitability of nociceptive neurons, amplifying nociceptive transmission and consequently enhancing pain perception.

Although numerous studies have demonstrated the involvement of several mechanisms associated with glial cells in central pain sensitization, they have been limited to the spinal level, and few studies have investigated this process in the thalamus, where central pain sensitization at the supraspinal level may also occur. As previously described, the VPL and VPM nuclei are involved in nociceptive synapses, which receive information from the spinothalamic tract and transmit it to the primary somatosensory cortex [12,13]. Since interactions between neurotransmitters, mediators of neurons, and glial cells also occur in this area, a “supraspinal” central sensitization can also occur in various types of pain, especially neuropathic pain.

An interesting clinical study conducted with seven individuals with peripheral nerve injury caused by limb amputation or brachial plexus injury found microglial activation in the contralateral VPL nucleus of the thalamus to the injury or amputation. This activation was verified by intravenous administration of the activated microglia marker (*R*)-PK11195, evaluated by positron emission tomography (PET) [66]. Zymosan-induced microglial activation and its response to minocycline can be quantitatively imaged in rats using the same technique [67]. In addition to microglia, an in vitro study found the involvement of astrocytes in the activation of neurons in the ventrobasal complex of the thalamus [68].

One of the initial studies conducted in rodents that evaluated this process also found an increased mRNA expression for the microglial activation marker IBA1 in the thalamus of mice. This effect was associated with a reduction in the mechanical nociceptive threshold after two weeks of inducing peripheral neuropathy by sciatic nerve compression [69]. Another similar study also found an increased gene expression of the respective microglial and astrocytic markers IBA1 and GFAP in the VPL nucleus in rats with neuropathic pain. In addition to glial cells, the authors demonstrated that brain-derived neurotrophic factor (BDNF) was also increased at this site [70]. Studies have already shown that BDNF participates in nociception at the spinal level, released by activated microglia [71,72]. Other studies have found microglia and astrocyte involvement in the VPL nucleus of the thalamus during neuropathic pain in rats [73,74]. In addition, the intracellular p38 MAPK signaling pathway has been implicated in neuropathic pain in rats [75]. Although these previous studies demonstrate the involvement of glial cells in some nuclei of the basal complex of the thalamus in modulating nociceptive impulses during neuropathic pain, the mechanisms involved in the activation of these cells, how they are activated, and how they can activate neurons at this site is still unclear. A recent study found increased TLR4 expression but observed no change in the expression of the transient receptor potential vanilloid type 1 (TRPV1) channel (an ion channel permeable to calcium ions) in microglia and astrocytes in the ventrobasal thalamic complex during neuropathic pain in mice. Additionally, the authors demonstrated the participation of the p38 MAPK pathway and the pro-inflammatory cytokine TNF-α in this process [76].

Recently, some studies have presented new mechanisms. However, they used a model of central neuropathic pain induced by intra-thalamic hemorrhage. Among the mechanisms, one study found the involvement of the P2X4 receptor expressed in microglia in the thalamus. In addition, FGR, a member of the Src-family nonreceptor tyrosine kinases, as well as the TLR4/NF-κB/ERK1/2/TNF-α pathway, have also been described as involved in central neuropathic pain induced by intra-thalamic hemorrhage [77,78,79]. Some authors demonstrated an overexpression in thalamic hypoxia-inducible factor-1α (HIF-1α) and NOD-like receptor thermal protein domain-associated protein 3 (NLRP3), which are associated with microglial and astrocytic activation followed by increased pro-inflammatory cytokine levels in the thalamus, and mechanical allodynia during central neuropathic pain [80,81]. Furthermore, siRNA HIF-1α or siRNA NLRP3 microinjection into the VPL nucleus before hemorrhage induction reversed mechanical allodynia. Lificiguat (YC-1) and MCC950 (respective inhibitors of HIF-1α and NLRP3) administration into the VPL of the thalamus 30 min before hemorrhage also reduced mechanical allodynia [80,81]. Reinforcing this finding, another study demonstrated that microRNA (miR)-223 can control intra-thalamic hemorrhage-induced neuropathic pain, an effect mediated by inhibition of NLRP3 expression. Furthermore, NLRP3 levels, pro-inflammatory cytokines, and mechanical allodynia were attenuated by miR-223 agomir injection [82]. Additionally, systemic pretreatment with ZL006, a small molecule that disrupts a protein–protein interaction between postsynaptic density protein 95 (PSD-95) and neuronal nitric oxide synthase (nNOS), alleviated the pain hypersensitivities in a dose-dependent manner, showing the relevance of a nitric oxide-mediated system in the control of central neuropathic pain at the thalamic level [83].

Another interesting study found a reduction of peroxisome proliferator-activated receptor gamma (PPARγ) in the VPM and VPL nuclei, which was co-localized with increased microglial expression in mice subjected to intra-thalamic hemorrhage. Furthermore, the PPARγ agonist pioglitazone alleviated mechanical allodynia, which was aggravated by its antagonist GW9662. Similar findings were found with anti-inflammatory cytokines in the thalamus of animals with central hemorrhage, which were upregulated by pioglitazone pretreatment and reduced by GW9662 administration. These findings demonstrated PPARγ importance in controlling central neuropathic pain at the thalamic level [84].

Panx1, a type of large-pore ion channel that can be expressed in microglia, was identified in participants with thalamic hemorrhage-induced central neuropathic pain. A study demonstrated that pretreatment with a selective Panx1 inhibitor attenuated mechanical allodynia. In addition, Panx1 inhibition was associated with reduced transcription of pro-inflammatory factors [85].

**Figure 3:**
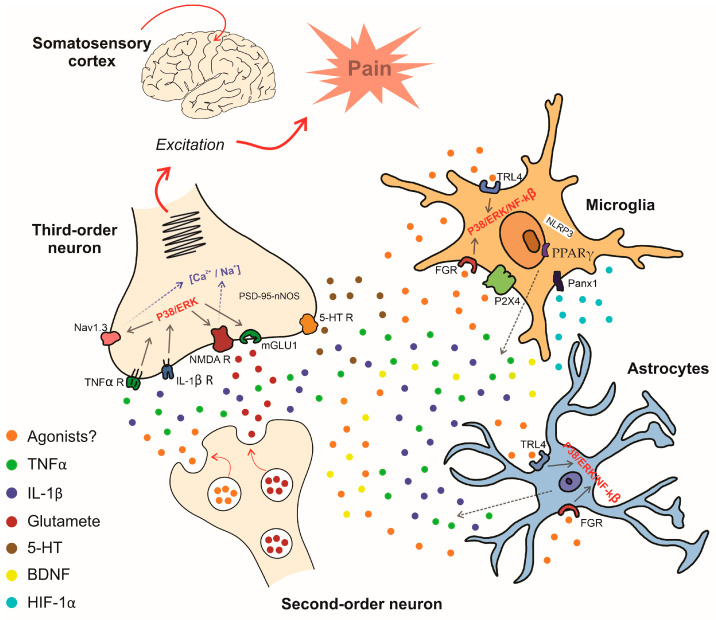
Possible mechanisms involved in the nociceptive synapse at the thalamic level. Stimulation of the third-order neuron by neurotransmitters released from the second-order neuron and its enhancement mediated by cytokines released by glial cells (see the text for details).

Another candidate involved in hemorrhage-induced thalamic pain is FTO (fat mass and obesity-associated protein), an N6-methyladenosine demethylase. The participation of this enzyme was verified after intraperitoneal administration of meclofenamic acid (an FTO inhibitor) or microinjection of adeno-associated virus 5 (AAV5) expressing Cre into the thalamus of Ftofl/fl mice, which attenuated TLR4 upregulation, thalamic damage, and mechanical allodynia [77]. These mechanisms are shown in Figure 3.

Although these studies described are crucial since they simulate a model of neuropathic pain that can often occur after a stroke or head trauma, they did not investigate new mechanisms involved in the participation of the thalamus during the physiology of chronic pain of peripheral origin, which has a high incidence worldwide.

The majority of the studies discussed showed various mechanisms involved in central pain sensitization, primarily in neuropathic pain. This type of pain involves abnormal signals or spontaneous neural activity, as well as increased sensitivity not only in injured axons but also in intact nociceptors. Overall, these events are linked to the upregulation or downregulation of different molecules in both the central and peripheral nervous systems. This characteristic makes neuropathic pain a more reproducible subject for study.

Furthermore, although the studies presented above have indicated some thalamic mechanisms involved during neuropathic pain, it is important to highlight that the thalamus is an important area involved in other types of pain, such as low back pain and cancer pain. Increased glial activity in the thalamus of patients with chronic low back pain was verified in studies using positron emission tomography, which detects [11C]-PBR28, a radioligand that binds to the 18 kDa translocator protein (TSPO), a mitochondrial molecule used as glial activation marker [86,87]. In a previous study investigating the effect of percutaneous ventrolateral cervical cordotomy in five patients with severe chronic pain due to cancer, positron emission tomography revealed reduced thalamic blood flow after this procedure. The authors suggested that this finding may be associated with decreased synaptic activity at the thalamic level, either due to the decreased activity of neurons projecting to that region and/or attenuated local neuronal firing, demonstrating the active participation of the thalamus in this type of pain [88]. Although the thalamus participates in nociceptive transmission in other chronic pain models in addition to neuropathic pain, more mechanisms need to be elucidated about this process.

As demonstrated in the present review, several studies utilizing preclinical models such as rats, mice, and monkeys have demonstrated the thalamus’s role in nociceptive transmission. These studies applied electrical stimuli and excitatory and inhibitory amino acids to block neurotransmission in the ventrobasal thalamic complex (VPL and/or VPM), evaluating mechanical and thermal nociceptive stimuli in neuropathic and inflammation-induced pain models.

While these preclinical studies are important in highlighting the thalamus’s role in nociceptive transmission, there are still many mechanisms that require further elucidation. Therefore, translational clinical studies are crucial to broaden the understanding of the thalamus’s involvement in chronic pain pathogenesis. This will contribute to the development of innovative and more efficient strategies for pain management.

## 6. Conclusions

Similar to the spinal dorsal horn, the thalamus is responsible for transmitting nociceptive impulses. In particular, the ventrobasal nuclei play a crucial role in relaying nociception to the somatosensory cortex. However, the mechanisms involved in this process are not well understood. This review highlighted the current knowledge about the thalamic mechanisms involved in the nociceptive process. Furthermore, some thalamic targets demonstrated here, such as NMDA, mGlu1, P2X4, NLRP3, TLR4, and FGR, in addition to microglia, astrocytes, and MAPKs, could be important for the development of more controlled mechanistic clinical studies and new therapies for pain control. Nevertheless, additional mechanisms await elucidation for a more comprehensive understanding of the processes involved.
